# Mapping of DNA methylation-sensitive cellular processes in gingival and periodontal ligament fibroblasts in the context of periodontal tissue homeostasis

**DOI:** 10.3389/fimmu.2023.1078031

**Published:** 2023-01-26

**Authors:** Katarzyna B. Lagosz-Cwik, Mariia Melnykova, Elwira Nieboga, Aureliusz Schuster, Agnieszka Bysiek, Slawomir Dudek, Weronika Lipska, Malgorzata Kantorowicz, Michal Tyrakowski, Dagmara Darczuk, Tomasz Kaczmarzyk, Marjolijn Gilijamse, Teun J. de Vries, Jan Potempa, Aleksander M. Grabiec

**Affiliations:** ^1^ Department of Microbiology, Faculty of Biochemistry, Biophysics and Biotechnology, Jagiellonian University, Kraków, Poland; ^2^ Department of Periodontology, Preventive Dentistry and Oral Medicine, Faculty of Medicine, Jagiellonian University Medical College, Kraków, Poland; ^3^ Chair of Oral Surgery, Faculty of Medicine, Jagiellonian University Medical College, Kraków, Poland; ^4^ Department of Oral and Maxillofacial Surgery and Oral Pathology, Amsterdam University Medical Center (Amsterdam UMC), Amsterdam, Netherlands; ^5^ Department of Oral and Maxillofacial Surgery, OLVG Hospital, Amsterdam, Netherlands; ^6^ Department of Oral Cell Biology, Academic Centre for Dentistry Amsterdam (ACTA), University of Amsterdam and Vrije Universiteit Amsterdam, Amsterdam, Netherlands; ^7^ Department of Oral Immunology and Infectious Diseases, University of Louisville School of Dentistry, Louisville, KY, United States

**Keywords:** periodontitis, gingival fibroblast, periodontal ligament fibroblast, DNA methyltransferases, decitabine (DAC), *Porphyromonas gingivalis*

## Abstract

Interactions between gingival fibroblasts (GFs) and oral pathogens contribute to the chronicity of inflammation in periodontitis. Epigenetic changes in DNA methylation are involved in periodontitis pathogenesis, and recent studies indicate that DNA methyltransferase (DNMT) inhibitors may protect against epithelial barrier disruption and bone resorption. To assess the impact of DNMT inhibition on GFs, cells were cultured with decitabine (5-aza-2’-deoxycytidine, DAC) for 12 days to induce DNA hypomethylation. We observed several potentially detrimental effects of DAC on GF biological functions. First, extended treatment with DAC reduced GF proliferation and induced necrotic cell death. Second, DAC amplified *Porphyromonas gingivalis*- and cytokine-induced expression and secretion of the chemokine CCL20 and several matrix metalloproteinases (MMPs), including MMP1, MMP9, and MMP13. Similar pro-inflammatory effects of DAC were observed in periodontal ligament fibroblasts. Third, DAC upregulated intercellular adhesion molecule-1 (ICAM-1), which was associated with increased *P. gingivalis* adherence to GFs and may contribute to bacterial dissemination. Finally, analysis of DAC-induced genes identified by RNA sequencing revealed increased expression of *CCL20*, *CCL5*, *CCL8*, *CCL13*, *TNF*, *IL1A*, *IL18, IL33*, and *CSF3*, and showed that the most affected processes were related to immune and inflammatory responses. In contrast, the genes downregulated by DAC were associated with extracellular matrix and collagen fibril organization. Our observations demonstrate that studies of DNMT inhibitors provide important insights into the role of DNA methylation in cells involved in periodontitis pathogenesis. However, the therapeutic potential of hypomethylating agents in periodontal disease may be limited due to their cytotoxic effects on fibroblast populations and stimulation of pro-inflammatory pathways.

## Introduction

Periodontitis is a chronic inflammatory diseases initiated by microbial imbalance (dysbiosis). In its severe form, periodontitis affects more than 10% of the population, causing an estimated loss of more than 150 billion EUR in Europe alone in 2018 (1). It is currently accepted that pathological changes in the gingival tissue are driven by the non-resolving host immune response to oral pathogens (2), among which *Porphyromonas gingivalis* plays a central role (3). Excessive production of inflammatory mediators by infiltrating immune cells and resident gingival cells drives connective tissue breakdown. As a consequence, chronic inflammation not only is the main cause of tissue damage and alveolar bone resorption, but also sustains dysbiotic microbiota by providing a source of nutrients for inflammophilic bacteria (4). Studies of individual cell populations in periodontitis have predominantly focused on gingival epithelial cells (GECs) as the natural first barrier protecting against oral pathogens (5). However, recent analysis of the human oral mucosal tissues at the single cell level identified immune functionality of stromal cells, in particular gingival fibroblasts (GFs) (6). Specifically, GFs play an essential role in recruiting neutrophils and lymphocytes into disease lesions (6).

Interactions with oral pathogens and the inflammatory tissue environment could lead to epigenetic changes in cells of the periodontium (7). Among the epigenetic regulatory mechanisms, methylation of cytosine residues at CpG dinucleotides is the most common and thoroughly characterized. Methylation marks on the DNA strand are established by DNA methyltransferases (DNMTs) in a site-specific manner and are typically associated with transcriptional repression due to chromatin condensation and/or disruption of interactions between transcription factors and DNA (8). While DNMT1 activity maintains DNA methylation profiles after replication, DNMT3a and DNMT3b are responsible for *de novo* DNA methylation (9). Facilitated by the rapid development of epigenomic technologies, dysregulated DNA methylation profiles, both globally and at individual gene promoters, have been identified in many pathologies, including inflammatory diseases and several types of cancer (10, 11). Importantly, aberrant DNA methylation can be targeted with small-molecule DNMT inhibitors. These compounds epigenetically reactivate silenced genes by inducing DNA hypomethylation and their potential to upregulate tumor suppressor genes has indicated their therapeutic potential in oncology. Indeed, 5-azacitidine (AZA) and 2’-deoxy-5-azacitidine (decitabine, DAC) are used for the treatment of acute myeloid leukemia (AML) and myelodysplastic syndrome (MDS) (12, 13). However, the use of these compounds in clinical practice has been restricted to oncology due to their significant hematologic toxicity (14).

In recent years, several studies have identified epigenetic regulatory mechanisms as key factors in periodontitis pathogenesis and possible targets for therapeutic strategies aimed at the modulation of host responses (15). Alterations in the methylation status of genes associated with immune responses, such as *IL6*, *IL8*, *IFNG*, *TNF*, *PTGS2*, and *TLR2*, have been identified in gingival biopsies collected from patients with periodontal disease (16). However, many of the reported differences were not reproducible in independent studies or their biological relevance has not been verified in functional analyses (7). In contrast, *in vitro* studies have provided more consistent results. Exposure of cells of the periodontium to oral pathogens or inflammatory cytokines tends to induce promoter-specific hypermethylation. In GECs, chronic infection with *P. gingivalis* caused hypermethylation of the TLR2 promoter (17), while extended treatment of periodontal ligament fibroblasts (PDLFs) with *P. gingivalis* lipopolysaccharide (LPS) increased the methylation levels of genes encoding extracellular matrix (ECM) components (18). These observations provided evidence that oral pathogens directly influence host responses through the modulation of DNA methylation and suggested that DNMT inhibitors may be therapeutically beneficial in the context of periodontitis pathogenesis. Indeed, in a mouse model of periodontitis, DAC suppressed osteoclastogenesis and upregulated anti-inflammatory cytokines, which resulted in reduced bone resorption (19). GEC treatment with DNMT inhibitors reversed *P. gingivalis*-induced functional impairment of the gingival epithelial barrier, which was associated with increased promoter methylation and expression level of genes encoding proteins that form cell-cell junction complexes (20). GF treatment with AZA promoted bone morphogenetic protein-2-induced differentiation of GFs into osteoblasts that were capable of inducing bone formation *in vivo* (21). These findings indicate that DNA hypomethylating agents may reverse the pathological effects of dysbiosis and promote the resolution of inflammation in periodontitis. However, the biological consequences of extended treatment with DNMT inhibitors on GF responses in the context of chronic inflammation and constant exposure to oral pathogens in the inflamed gingival tissue remain unknown. In this study, we comprehensively analyzed the transcriptional and functional consequences of primary human GF and PDLF treatment with DNMT inhibitors, identifying several key cellular process that are affected by DNA hypomethylation.

## Materials and methods

### Subjects and cell isolation

Gingival tissue specimens were collected from 16 healthy individuals undergoing orthodontic treatment at the Department of Periodontology and Clinical Oral Pathology and Chair of Oral Surgery, Faculty of Medicine, Jagiellonian University Medical College, Krakow, Poland. Teeth were collected from five patients referred for multiple adjacent tooth extractions due to gingivitis, periodontitis, or tooth decay at the Department of Oral and Maxillofacial Surgery at the OLVG hospital in Amsterdam, The Netherlands. GFs and PDLFs were isolated from gingival biopsies and teeth, respectively, according to the previously described protocols (22, 23), cultured in DMEM (Lonza) containing 10% fetal bovine serum (FBS) (Biowest), gentamicin (50 U/ml) and penicillin/streptomycin (50 U/ml) (both from Gibco), and used for experiments between passages 4 and 9.

### Bacterial culture

Wild-type *P. gingivalis* (strain ATCC33277) were grown anaerobically on blood agar plates for 5-7 days before inoculation into brain-heart infusion (BHI) broth (Becton-Dickinson) with yeast extract containing 10 µg/mL hemin, 0.5 mg/mL L-cysteine, and 0.5 µg/mL vitamin K. Bacterial suspensions at optical density (OD)_600_ = 1 (corresponding to 10^9^ colony-forming units (CFU)/ml) were prepared as described before (23).

### Cell culture, treatment with DNMT inhibitors, stimulation, and infection

One day prior to treatment with inhibitors, cells were seeded in 10 cm dishes in medium supplemented with 10% FBS and antibiotics. Cells were cultured for 12 days in the presence of DMSO (control) or DNMT inhibitors: decitabine (DAC; 5 μM), 5-azacitidine (AZA; 5 μM) and 6-thioguanine (6-TG; 10 μM) (all from TargetMol). Every 4 days, the medium was replaced, and fresh portion of inhibitors was added. Alternatively, GFs were treated with DMSO or DAC for 12 days, with replacement of medium and compounds every day. In an independent set of experiments, GFs were cultured in the presence of DMSO or DAC for 3 days, either receiving a single dose of the compounds during treatment, or fresh portions of medium with DMSO or DAC every day. Cells were then split, counted, and seeded for infection or cytokine stimulation as described below.

### Cell proliferation and viability assays

Cell proliferation was assessed using a Cell Proliferation ELISA BrdU kit (Roche). DAC- or DMSO-treated GFs were seeded in 96-well plates and after overnight culture a BrdU labeling reagent was added. Cells were then cultured for 24 h, fixed and incubated with anti-BrdU-POD antibody for 90 min. A Cytotoxicity Detection Kit (LDH) and Cell Death Detection ELISA (both from Roche) were used to assess cytotoxic and proapoptotic effects of DNMT inhibitors. After culture with DMSO or DAC, GFs were seeded in 96-well plates and cultured for 24 h. Staurosporine (STS, 1 µM) was used as a positive control for cell death induction. Next, supernatants were collected and the assays were performed according to the manufacturers’ protocols. For the LDH assay, supernatants from lysis buffer-treated cells were used as a reference displaying 100% cytotoxicity. A FlexStation3 Multi-Mode microplate reader (Molecular Devices) was used for absorbance measurements.

### ELISA

GFs or PDLFs seeded in 96-well plates were infected with *P. gingivalis* (MOI 10 or 50) for 1 h followed by 23 h of culture in fresh medium, or were stimulated with tumor necrosis factor (TNF) or IL-1β (both from BioLegend) at 10 ng/ml for 24 h. Concentrations of analytes were determined in cell-free supernatants using a CCL2 ELISA MAX Standard sets (BioLegend) or CCL20 and MMP1 ELISA Duo Sets (R&D Systems), according to the manufacturers’ instructions.

### RNA isolation and quantitative polymerase chain reaction (qPCR)

Total RNA was extracted using an ExtractMe Total RNA isolation kit (Blirt) and quantified using a BioPhotometer D30 (Eppendorf). A High-Capacity cDNA Reverse Transcription Kit (Applied Biosystems) was used for RNA conversion to cDNA. qPCR reactions were performed on a CFX96 Touch™ Real-Time PCR Detection System (Bio-Rad) using PowerUp SYBR Green PCR mix (Applied Biosystems). The CFX Manager (Bio-Rad) was used for data analysis. mRNA expression relative to a housekeeping gene (*RPLP0*) was calculated using the ΔΔCT method. The sequences of the primers used in the study (purchased from Merck) are listed in **Supplementary Data Sheet 1: Table S1**.

### Immunoblotting

Protein expression in cell lysates was determined by immunoblotting, as described previously (24) using antibodies recognizing ICAM-1 (#67836) or β-actin (#4967) (both from Cell Signaling Technology). A ChemiDoc MP Imaging System (Bio-Rad) was used for membrane visualization.

### DNA isolation and analysis of global DNA methylation

Genomic DNA was extracted using a DNeasy Blood and Tissue Kit (Qiagen) and quantified with a BioPhotometer D30. Global DNA methylation was assessed using a Global DNA Methylation LINE-1 Kit (Active Motif), according to the protocol provided by the manufacturer. Briefly, 1 μg of DNA from each sample was digested with *MseI* overnight at 37°C, and then the reaction was stopped by incubation of the samples at 60°C. Next, DNA was diluted to a final concentration 4 ng/ml and incubated with the LINE-1 Probe Solution. Samples and standards were then transferred to streptavidin-coated plates and incubated with a 5-methylcytosine antibody. Absorbance was measured at 450/655 nm and % 5-mC of CpG residues relative to the total cytosine content was determined.

### Colony-forming assay

After 12-day culture with DMSO or DAC, GFs were seeded in duplicate wells in 12-well plates (10^5^ cells per well) and infected with *P. gingivalis* (MOI 100) for 1 h. To discriminate between adhesion and internalization of bacteria, cells were washed three times with PBS and cultured for 1 h either in antibiotic-free fresh medium, or in medium with gentamicin (2.5 mg/ml) and metronidazole (2 mg/ml) for 1 h. Next, cells were lysed in sterile, distilled water for 40 min, 10-fold serial dilutions of cell lysates were prepared and 10 µl of each dilution was transferred to BHI blood agar plates in duplicate. *P. gingivalis* colonies were counted after plate incubation for 5 days at 37°C under anaerobic conditions.

### Analysis of CCL20 degradation by *P. gingivalis*

*P. gingivalis* at 10^6^, 2.5×10^6^, 5×10^6^ and 10^7^ CFU/ml was incubated with 10 ng/ml CCL20 (BioLegend) in DMEM containing 2% FBS without antibiotics. Supernatants were collected after 2 h and 24 h and centrifuged to remove bacteria (10 000×g, 10 min, 4°C CCL20 concentrations were determined by ELISA.

### *P. gingivalis* growth curve analysis

*P. gingivalis* cultures were inoculated into BHI broth containing 0.5 mg/ml L-cysteine, 10 µg/ml hemin and 0.5 µg/ml vitamin K at OD_600_ = 0.1. Bacterial suspensions were cultured anaerobically either with recombinant human CCL20 (BioLegend) at 0.2 or 1 µg/ml, or with bestatin (5 mg/ml) which was used as a control compound that inhibits the growth of *P. gingivalis*. Bacterial growth was determined by OD measurements at 0, 4, 8, and 24 h using a Cell Density Meter (Biochrom).

### RNA sequencing

After 12-day culture with DMSO or DAC, GFs (n=5) were seeded in 6-well plates (5×10^5^ cells per well) for 24 h and then were either left untreated or were infected with *P. gingivalis* (MOI 20) for 4 h. Quality of RNA extracted using an RNeasy Mini Kit (Qiagen) was assessed using an RNA Nano 6000 Assay Kit of the Bioanalyzer 2100 system (Agilent Technologies). Library preparation, clustering, sequencing and data processing and analysis were performed at Intelliseq S.A. (Krakow, Poland) and are described in detail in **Supplementary Data Sheet 1: Supplementary Materials and Methods**.

### Statistical analyses

Data are presented as the mean +SEM. Primary cells isolated from different donors were used in all experiments and the values of ‘n’ indicate the number of cell lines derived from independent donors used in each experiment. The ratio paired *t*-test was used for comparisons between groups unless otherwise indicated. *P* values <0.05 were considered statistically significant.

## Results

### DNMT inhibition reduces GF proliferation and viability

**Figure 1:**
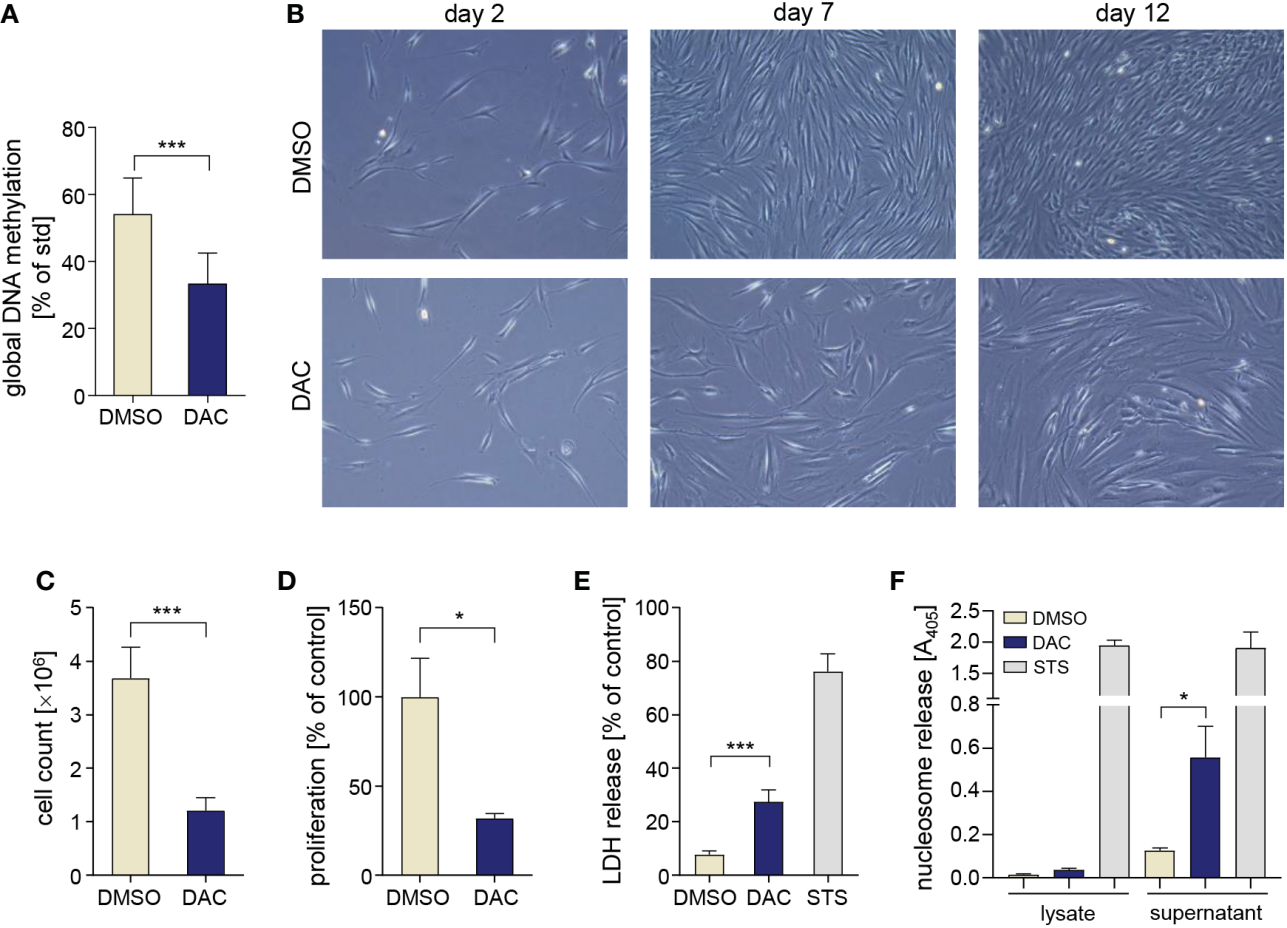
DAC induces global DNA hypomethylation and has anti-proliferative and cytotoxic effects on GFs. **(A)** Global DNA methylation in GFs cultured with DMSO or DAC for 12 days. **(B)** Representative micrographs of GFs cultured in the presence of DMSO or DAC for 12 days. Pictures were taken on days 2, 7, and 12 of the experiment using contrast-phase microscopy. **(C)** GF total cell counts (n=4) and **(D)** proliferation rate determined using the BrdU incorporation assay (n=8) after 12 days of culture with DMSO or DAC. Viability of GFs treated with DMSO or DAC for 12 days measured using **(E)** the LDH release assay (n=7) and **(F)** analysis of nucleosome release into cytosolic cell fractions (n=3) and culture supernatants (n=4). GFs treated with 1 µM staurosporine (STS) were used as a positive control. *P < 0.05, ***P < 0.001.

To verify how DNMT inhibition affects key biological functions of gingival stromal cells, we cultured primary human GFs with 5 µM DAC for 12 days to induce DNA hypomethylation. This experimental setup allows for multiple cell divisions that are required for efficient erasing of DNA methylation patterns that are not maintained during replication in the absence of DNMT1 activity. GF culture in the presence of DAC caused a 40% reduction of global DNA methylation (**Figure 1A**). During GF culture, we noted reduced density of cells that were cultured with DAC on day 7 and day 12 (**Figure 1B**), which was associated with significantly lower numbers of DAC-treated cells that were retrieved from culture dishes compared to DMSO-treated cells (**Figure 1C**). Reduction of GF cell numbers during culture with DAC was caused by reduced proliferation (**Figure 1D**) as well as cytotoxic effects of the DNMT inhibitor (**Figure 1E**) as determined by BrdU incorporation and the LDH release assay, respectively. The cytotoxic and anti-proliferative effects of DAC were dose-dependent and readily detectable upon GF treatment with 0.2 µM DAC (**Supplementary Data Sheet 1: Figures S1A, B**). Next, we analyzed the presence of nucleosomes in the cytosol and culture supernatants to identify the type of cell death induced by DAC in GFs. While DAC treatment did not cause the accumulation of nucleosomes in the cytosol, which is a hallmark of apoptosis, we detected significant levels of DNA-complexed histones in the supernatants, indicative of cell necrosis (**Figure 1F**). These results indicate that extended treatment with the DNMT inhibitor DAC has detrimental effects on GF proliferation and viability.

### DNMT inhibitors promote the production of CCL20 and MMPs by GFs

**Figure 2:**
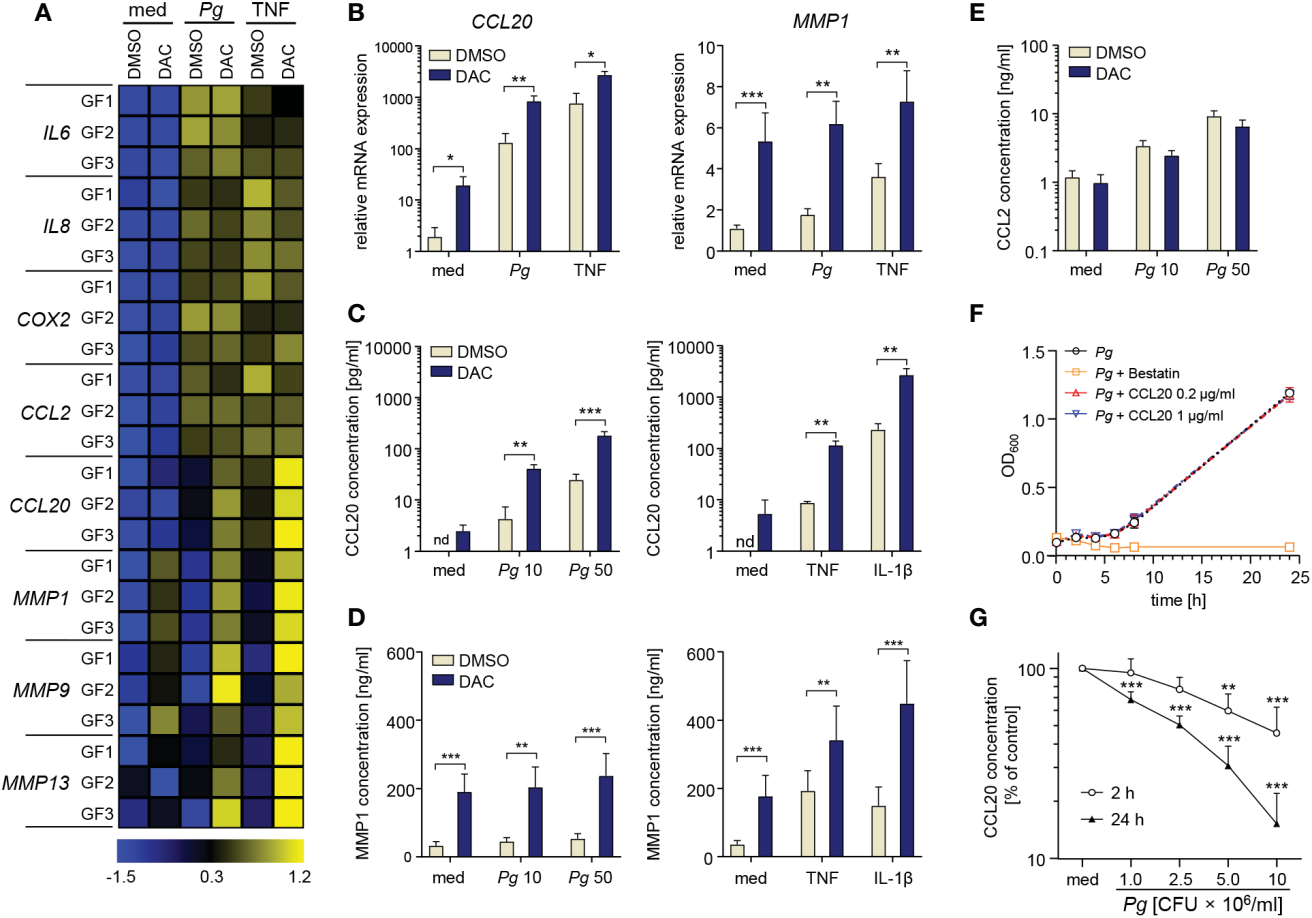
DAC promotes production of CCL20 and MMPs by GFs. **(A, B)** GFs treated with DMSO or DAC for 12 days were left unstimulated in medium (med), or were infected with *P. gingivalis* (*Pg*, MOI 20), or stimulated with TNF (10 ng/ml) for 4 h. **(A)** qPCR analysis of mRNA expression of inflammatory mediators in GFs from individual donors (GF1-GF3) shown on a heatmap as row Z-scores calculated from ΔCt values relative to a housekeeping gene (*RPLP0*). **(B)** Relative mRNA levels of *CCL20* and *MMP1* (n=5). **(C-E)** DMSO- or DAC-treated GFs were infected with *P. gingivalis* (MOI 10 or 50) for 1 h followed by washing and culture for another 23 h in fresh medium containing antibiotics or were stimulated with TNF or IL-1β (both 10 ng/ml) for 24 h. Production of **(C)** CCL20, **(D)** MMP1 and **(E)** CCL2 is shown as mean concentration +SEM (n=4-8). **(F)** Growth curve of *P. gingivalis* cultured anaerobically in the presence of recombinant human CCL20 (0.2 and 1 μg/ml) or bestatin (5 mg/ml) (positive control) (n=3). **(G)** Degradation rate of CCL20 by *P. gingivalis* at 10^6^, 2.5×10^6^, 5×10^6^, and 10^7^ CFU/ml after 2 h and 24 h of incubation (n=4). nd, not detectable; *P < 0.05, **P < 0.01, ***P < 0.001.

Next, we determined the effects of DNA hypomethylation on GF inflammatory activation. After 12 d of culture in the presence of DMSO or DAC, equivalent numbers of GFs were plated and infected with *P. gingivalis* or stimulated with TNF. GF culture with DAC had no effect on both basal and inducible expression of *IL6*, *IL8*, *COX2*, and *CCL2* mRNA (**Figure 2A**). In contrast, transcript levels of the chemokine CCL20 induced by *P. gingivalis* infection or TNF stimulation were significantly higher in DAC-treated cells. Similarly, both basal and inducible mRNA expression of several MMPs, including *MMP1*, *MMP9* and *MMP13* were elevated in DAC-treated cells (**Figures 2A, B**). Strikingly, despite reduced cell viability, DAC-mediated induction of CCL20 and MMP1 was even more pronounced at the protein level. Compared to cells cultured with DMSO, GFs cultured in the presence of DAC released at least 10-fold higher amounts CCL20 upon infection with *P. gingivalis* or stimulation with TNF or IL-1β (**Figure 2C**). The augmentation of TNF-induced CCL20 production by GFs was also apparent at lower DAC concentrations (**Supplementary Data Sheet 1: Figure S1C**). Similarly, the levels of MMP1 produced by GFs cultured with DAC were significantly higher compared to DMSO-treated cells regardless of the absence or presence of cytokine stimulation or bacterial infection (**Figure 2D**). Consistent with mRNA expression data, secretion of CCL2 was largely unaffected by DAC (**Figure 2E**).

While the detrimental effects of MMPs in periodontitis have been well-documented (25), the role of CCL20 is controversial due to its dual biological activity as a chemokine and an antimicrobial peptide (26). To verify whether increased amounts of CCL20 produced by DAC-treated GFs could be sufficient for direct microbicidal effects, we cultured *P. gingivalis* anaerobically in the presence of recombinant CCL20 or bestatin as a control known to inhibit *P. gingivalis* growth (27). While bestatin had a strong bacteriostatic effect, CCL20 at either tested concentration had no effect on *P. gingivalis* proliferation (**Figure 2F**). The inability of CCL20 to affect *P. gingivalis* growth, in contrast to its antimicrobial activity on other Gram-negative bacteria (28), may be due to degradation by bacterial proteases, among which gingipains play the most prominent role (29). Indeed, incubation of recombinant CCL20 with *P. gingivalis* in conditions equivalent to those used in cell infection experiments resulted in rapid degradation of the protein (**Figure 2G**). These data indicate that CCL20 does not exert a direct antimicrobial effect on *P. gingivalis*. Therefore, increased production of CCL20 by DNMT-inhibitor treated GFs will likely have proinflammatory effects due to the recruitment of leukocytes. Collectively, these observations identify a cluster of inflammatory mediators that are dynamically regulated by changes in DNA methylation and suggest that DNMT inhibitors may amplify chronic inflammation in gingival tissue.

### Degradation products or off-target effects are not responsible for DAC effects on GFs

**Figure 3:**
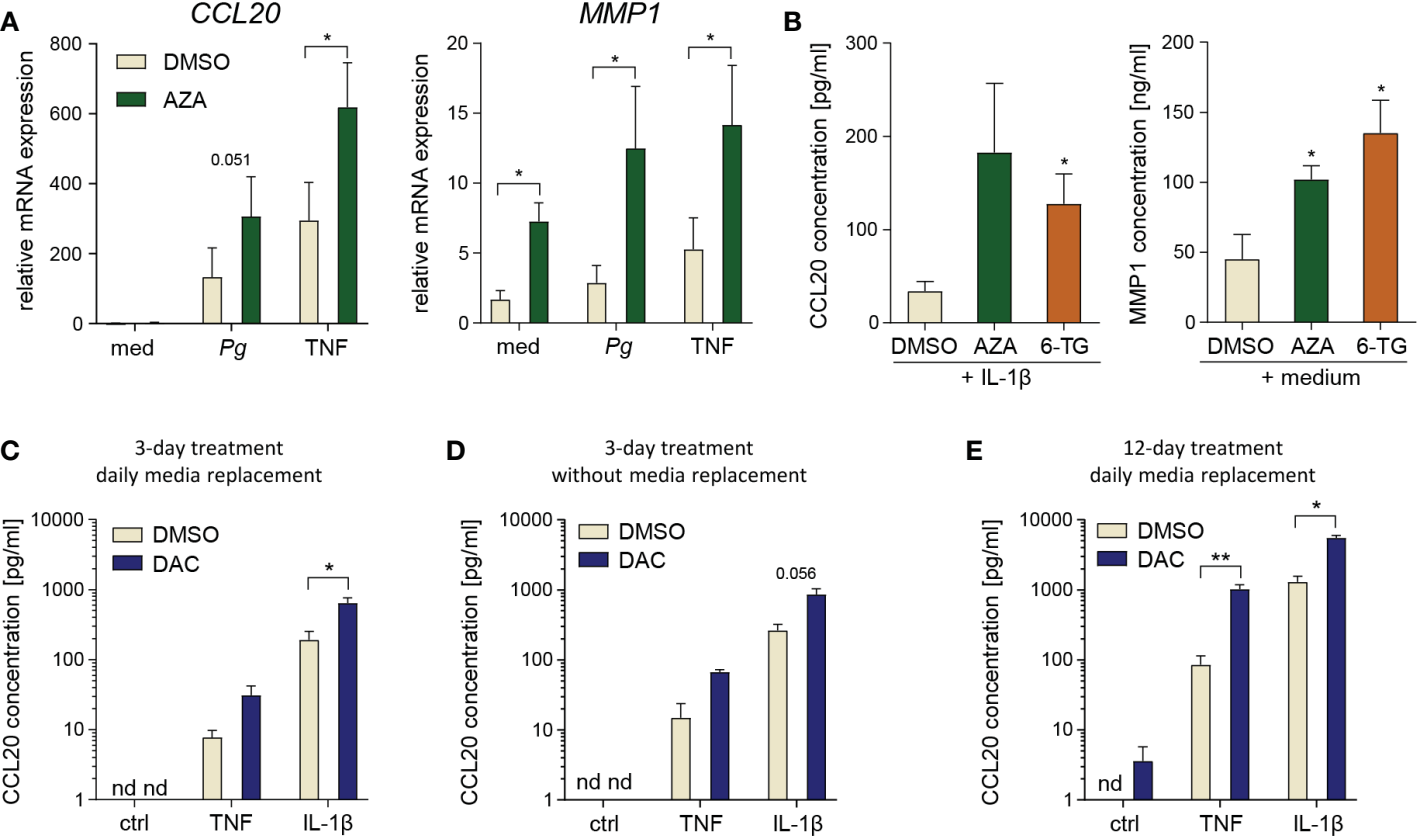
Degradation products or off-target effects are not responsible for DAC effects on GFs. **(A)** qPCR analysis of *CCL20* and *MMP1* mRNA expression in GFs treated with DMSO or 5-azacitidine (AZA) for 12 days followed by infection with *P. gingivalis* (*Pg*, MOI 20) or stimulation with TNF (10 ng/ml) for 4 h (n=5). **(B)** CCL20 and MMP1 production by GFs cultured with DMSO, AZA or 6-thioguanine (6-TG) prior to 24 h culture in medium alone or stimulation with IL-1β at 10 ng/ml h (n=5). *P < 0.05, RM One-way ANOVA followed by Bonferroni multiple comparison test. **(C-E)** CCL20 production by GFs (n=3-4) stimulated with TNF or IL-1β (both at 10 ng/ml) for 24 h after culture in the presence of DMSO or DAC: **(C)** for 3 days with replacement of medium and compounds every day, **(D)** for 3 days without replacement of medium and compounds, or **(E)** for 12 days with replacement of medium and compounds every day. nd, not detectable; *P < 0.05, **P < 0.01.

To exclude the possibility that the effects of DAC may be caused by off-target activity of the inhibitor, we cultured GFs with another cytidine analogue, AZA, and the structurally unrelated compound 6-TG. *P. gingivalis*- and TNF-induced *CCL20* and *MMP1* expression levels were significantly increased in AZA-treated cells (**Figure 3A**) and both compounds enhanced basal MMP1 levels and IL-1β-induced production of CCL20 by GFs (**Figure 3B**).

DAC has a half-life of approximately 11 h at physiological temperature and pH, and decomposes into several degradation products, some of which display toxic properties independent of the parent compound (30). Therefore, we next tested whether the elimination of these DAC degradation products, by replacing culture media every day during treatment and/or by reducing the culture time to 3 days, would limit the detrimental effects of DAC on GF survival. Although a 20% reduction in GF proliferation was observed upon 3-day DAC treatment with daily change of culture media (**Supplementary Data Sheet 1: Figure S2A**), this effect was not statistically significant and was not accompanied by increased LDH release (**Supplementary Data Sheet 1: Figure S2B**). In contrast, GF treatment with DAC for 3 days without daily replacement of culture media caused a more pronounced reduction of cell proliferation and a clear increase in LDH release (**Supplementary Data Sheet 1: Figures S2C, D**). Interestingly, 3-day treatment of GFs with DAC, with or without daily replacement of culture media, was sufficient to increase cytokine-induced CCL20 protein production (**Figures 3C, D**). However, these effects were less pronounced compared to those observed after 12-day cell exposure to DAC (**Figures 2B, C**). We also tested whether replacement of media with DAC every day during 12-day culture would limit the cytotoxicity of the compound. In this experimental protocol, the anti-proliferative and cytotoxic effects of DAC were comparable to those observed without daily change of DAC-containing media (**Supplementary Data Sheet 1: Figures S2E, F**), and so was the augmentation of cytokine-induced CCL20 production (**Figure 3E**). These results indicate that, whereas the early cytotoxic and anti-proliferative effects of DAC may be partly mediated by its degradation products, the long-term effects of DAC on GF viability occur despite regular removal of these products. Therefore, increased CCL20 production, as well as the cytotoxic effects observed after extended GF exposure to DAC, are likely a direct consequence of DNA hypomethylation induced by DAC and the contribution of compound decomposition products may be negligible.

### DNMT inhibition promotes *P. gingivalis* adhesion to GFs

**Figure 4:**
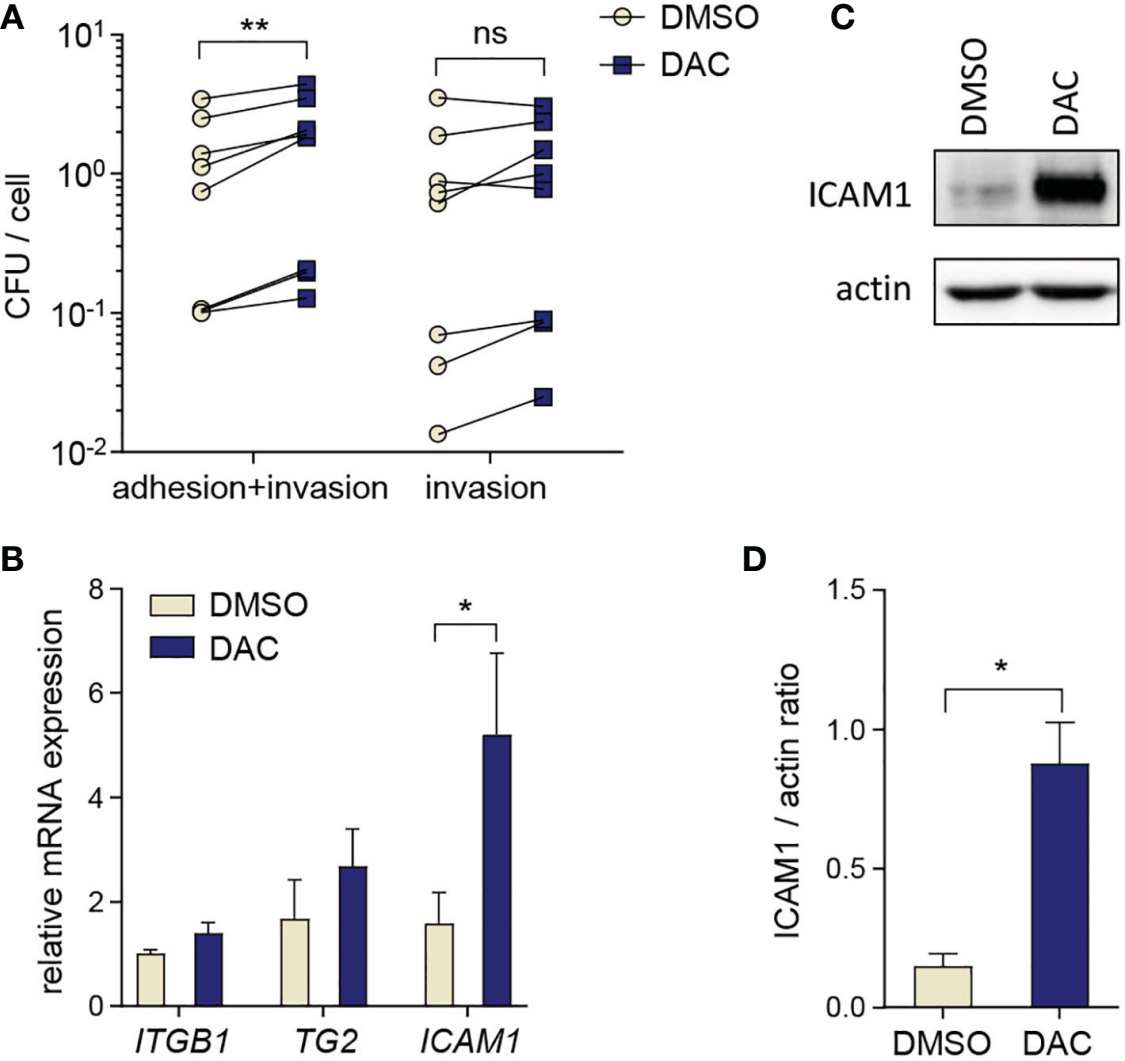
DAC promotes *P. gingivalis* adhesion to GFs. **(A)** Bacterial adhesion and intracellular survival determined by the colony forming assay in GFs (n=8) treated for 12 days with DMSO or DAC. Individual values of CFU counts per cell with lines connecting DMSO- and DAC-treated cells from individual donors are shown. **(B)** qPCR analysis of *ITGB1*, *TG2* and *ICAM1* in DMSO- and DAC-treated GFs (n=5). **(C, D)** Western blot analysis of ICAM1 and actin in GFs after 12 days of culture with DMSO or DAC. A representative of 4 independent experiments **(C)** and the ratio of ICAM1/actin signal intensity from densitometric analysis (n=4) **(D)** are shown. ns, not significant; *P < 0.05, **P < 0.01.

To gain more insight into the influence of DNA hypomethylation on *P. gingivalis* interactions with host cells, we analyzed *P. gingivalis* adhesion to and intracellular survival in GFs treated with DAC for 12 days. DAC treatment had no significant effect on the numbers of live *P. gingivalis* detected inside the cells after antibiotic treatment which removes adherent bacteria (**Figure 4A**). However, we noted increased CFU counts in DAC-treated GFs cultured without antibiotics after infection. This result reflects the combination of bacteria that adhered to and entered the cells and suggests that DAC treatment may promote bacterial adhesion to GFs (**Figure 4A**). To verify whether this effect may be caused by changes in expression of proteins that are involved in *P. gingivalis* adhesion to host cells (31, 32), we analyzed mRNA levels of integrin subunit β1 (*ITGB1*), transglutaminase-2 (*TG2*), and intercellular adhesion molecule-1 (*ICAM1*). Among these genes, only ICAM1 expression was significantly elevated in DAC-treated GFs (**Figure 4B**). Induction of ICAM1 expression by DAC was also evident at the protein level: GFs cultured with DAC expressed 6-fold higher levels of the ICAM-1 protein compared to cells treated with DMSO (**Figures 4C, D**).

### Global transcriptomic analysis of DAC-treated GFs identifies cellular processes affected by DNMT inhibition in GFs

**Figure 5:**
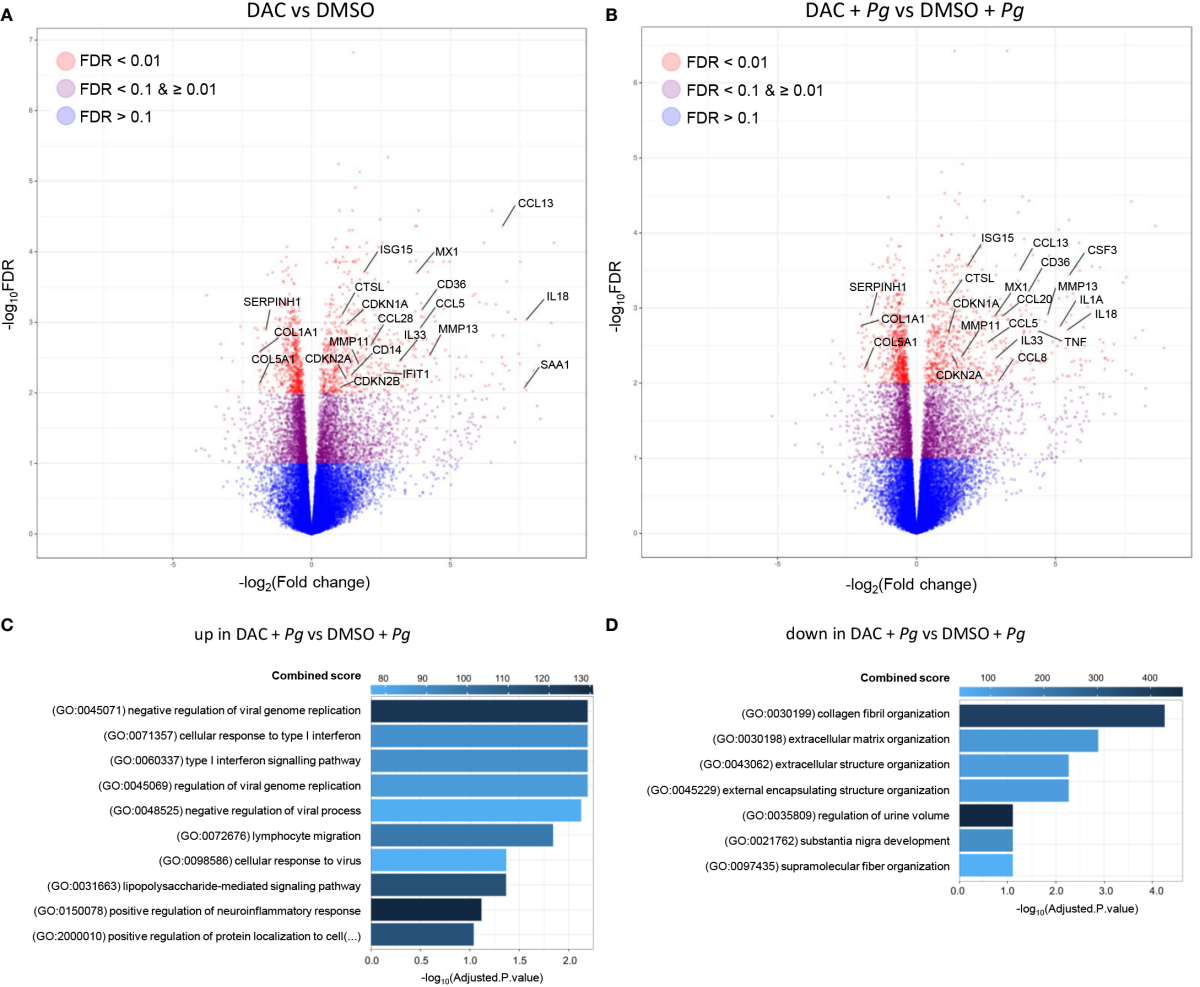
DAC induces global transcriptional changes in GFs. GFs (n=5) treated with DMSO or DAC for 12 days were left unstimulated in medium or were infected with *P. gingivalis* (*Pg*, MOI 20) for 4 h and mRNA expression was profiled by next generation sequencing. **(A, B)** Volcano plots of RNA-seq data showing the log_2_FC and -log_10_(FDR) of DMSO- and DAC-treated GFs in the absence **(A)** and presence **(B)** of *P. gingivalis* infection. **(C, D)** Pathway enrichment analysis of significantly upregulated **(C)** and downregulated **(D)** genes in DAC-treated GFs infected with *P. gingivalis*. Top hits based on Combined Score and adjusted *P*-value < 0.1 are presented.

To obtain a complete overview of cellular processes affected by DNMT inhibition, we performed a global transcriptomic analysis of GFs cultured with DMSO or DAC for 12 days prior to *P. gingivalis* infection. DAC induced major transcriptional changes: expression of approximately 500 genes was significantly modulated in DAC-treated cells compared to cells treated with DMSO both in the presence and absence of subsequent infection with *P. gingivalis* (**Figures 5A, B** and **Supplementary Table 2**). Nearly 80% of the affected genes were upregulated, consistent with the notion that DNA hypomethylation is typically associated with increased gene expression. Pathway analysis of significantly upregulated genes in DAC-treated GFs infected with *P. gingivalis* (including *CCL20*, *CCL5*, *CCL8*, *CCL13*, *TNF*, *IL1A*, *IL18, IL33*, and *CSF3*) showed that the most affected processes were related to immune and inflammatory responses. Interestingly, apart from lymphocyte migration and LPS signaling, several genes related to type I interferon signaling and responses to viral infection were upregulated after GF culture with DAC (*APOBEC3C*, *IFITM1*, *RSAD2*, *IFI27*, *OAS2*, *MX1*, *IFI6*, *ISG15*, *SAMHD1*) (**Figure 5C**). In contrast, the genes significantly downregulated by DAC were predominantly associated with the ECM and collagen fibril organization (*COL1A1*, *COL5A1*, and *SERPINH1*) (**Figure 5D**). These results not only substantiate but also extend our previous findings that the overall effect of DNMT inhibitors on GFs may be detrimental in the context of periodontitis pathogenesis due to the stimulation of inflammatory processes and dysregulation of ECM homeostasis. At the same time, the transcriptomic data reveal how broadly changes in DNA methylation affect key cellular processes in GFs.

### DMNT inhibition has anti-proliferative and proinflammatory effects in PDLFs

**Figure 6:**
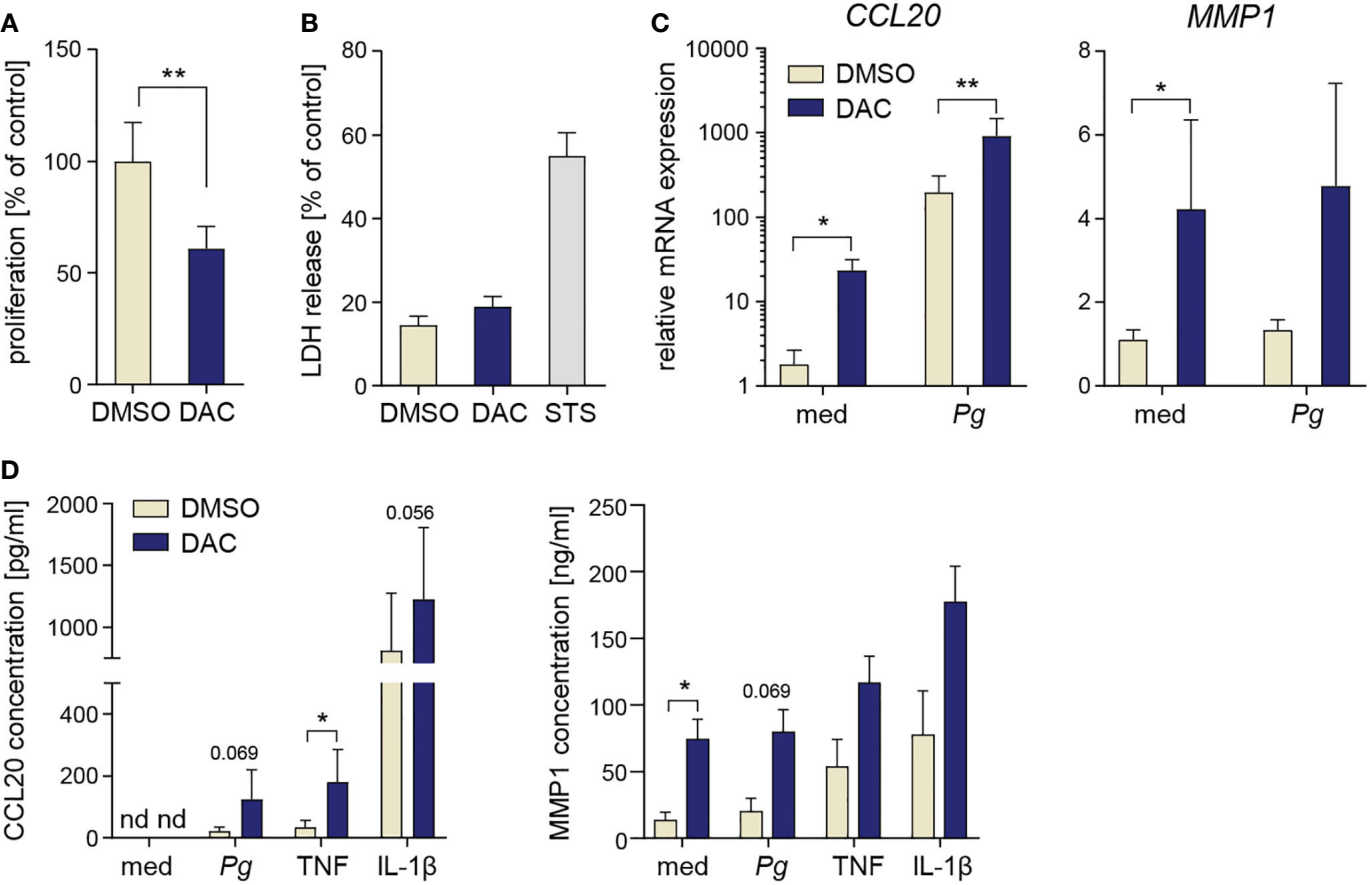
DAC has anti-proliferative and pro-inflammatory effects on PDLFs. **(A)** Proliferation rate (n=5) and **(B)** LDH release (n=4) in PDLFs treated with DMSO or DAC for 12 days. **(C)** qPCR analysis of *CCL20* and *MMP1* mRNA expression in PDLFs (n=5) treated with DMSO or DAC for 12 days prior to infection with *P. gingivalis* (*Pg*, MOI 20) for 4 h; ‘med’ refers to uninfected control. **(D)** CCL20 and MMP1 production by DMSO- or DAC-treated PDLFs that were infected with *P. gingivalis* (MOI 50) for 1 h followed by 23 h of culture in fresh medium, or were stimulated with TNF or IL-1β (both at 10 ng/ml) for 24 h (n=4). nd, not detectable; *P < 0.05, **P < 0.01.

Finally, we tested whether DNMT inhibition has similar effects on other stromal cells of the periodontium using PDLFs as a model. DAC significantly reduced PDLF proliferation (**Figure 6A**), but caused only a minor increase in LDH release (**Figure 6B**), suggesting that, in contrast to GFs, PDLFs are more resistant to the cytotoxic activity of DAC (or its metabolic products). After 12-day culture of PDLFs with DAC, both basal and inducible mRNA expression levels of CCL20 and MMP1 were significantly increased (**Figure 6C**). In line with the mRNA data, trends towards elevated CCL20 and MMP1 production by DAC-treated PDLFs were observed after cytokine stimulation or *P. gingivalis* infection, though the differences reached statistical significance only in some of the tested conditions (**Figure 6D**). Collectively, these results demonstrate that the potentially detrimental effects of DNMT inhibition on cell proliferation and inflammatory responses are not restricted to GFs and at least partly occur in other periodontium-derived stromal cells, such as PDLFs.

## Discussion

As one of the most prevalent chronic diseases, periodontitis represents a significant burden for healthcare systems (33). Uncontrolled inflammation of the periodontium not only causes tooth loss, but also leads to an increased risk of developing many systemic diseases, including rheumatoid arthritis, atherosclerosis, (RA), and malignancy (34, 35). Since the currently used treatment strategies focusing on reducing the bacterial challenge may be insufficient to restore microbial homeostasis and resolve chronic inflammation, there is a need for identifying new therapeutic options for the management of periodontitis (2). The idea of “host modulation therapy”, where medications aimed at the damaging aspects of the immune response support conventional periodontal treatment, has received much attention in recent years (36). Based on their ability to suppress inflammation and modulate host responses to pathogenic bacteria (37, 38), many epigenetic drugs have been evaluated in preclinical models of periodontitis. We have previously demonstrated that histone deacetylase inhibitors (HDACi) and BET bromodomain inhibitors suppress the inflammatory activation of GFs without affecting cell viability and susceptibility to bacterial invasion (23, 39). These observations, in combination with previous evidence that BET inhibitors and HDACi reduce inflammation and ameliorate alveolar bone resorption in animal models of periodontitis (40, 41), identified histone acetylation as a potential target for epigenetic host modulation therapy (7). In the present study, we show that major transcriptional changes induced by DNMT inhibitors affect the viability and inflammatory activation of GFs and PDLFs in ways that can be detrimental in the context of the pathogenesis of periodontitis. In light of the well-documented hematologic toxicities of hypomethylating agents (14), recent evidence of DAC genotoxicity (42), and our observations reported here, these compounds are unlikely candidates for host modulation therapy in periodontitis.

Nonetheless, our observations reveal the scope of cellular process in GFs that are regulated by DNA methylation. Among the 400 genes significantly upregulated in DAC-treated GFs infected with *P. gingivalis*, we identified several pro-inflammatory cytokines and chemokines, including *CCL20*, *CCL5*, *CCL8*, *CCL13*, *TNF*, *IL1A*, *IL18, IL33*, and *CSF3*. Elevated levels of many of these mediators have been detected in gingival crevicular fluid or tissue from patients with periodontitis (43), and their activity contributes to the chronicity of inflammation and alveolar bone resorption. For example, the levels of IL-33 and granulocyte colony-stimulating factor (G-CSF), encoded by the *CSF3* gene, are increased in mice with experimentally induced periodontitis and in gingival epithelial cells from periodontitis patients (44–46). *In vivo* experiments confirmed the pathological roles of these cytokines: treatment of mice with IL-33 exacerbates bone loss (44), whereas neutralization of G-CSF with a blocking antibody alleviates bone resorption (46). While our *in vitro* observations are not consistent with the study of Tanaka et al., who demonstrated immunomodulatory effects of DAC in experimental periodontitis (19), it should be noted that the influence of DNMT inhibitors on inflammatory processes is highly disease- and cell type-specific. DAC has been shown to promote cytokine production by human dental pulp cells and bronchial epithelial cells (47, 48), and upregulate pro-inflammatory pathways in an epithelial breast cancer cell line (49). In contrast, DAC treatment had anti-inflammatory effects in mouse models of atherosclerosis (50), acute respiratory distress syndrome (51), and arthritis (52).

The combination of transcriptomic and functional studies allowed us to identify multiple aspects of GF biology other than inflammatory pathway activation that are regulated by DNA methylation. Although pathway analysis did not reveal gene clusters involved in the regulation of cell survival, DAC treatment significantly upregulated cyclin-dependent kinase inhibitors *CDKN1A* (p21^CIP1^), *CDKN2A* (p16^INK4a^), and *CDKN2B* (p15^INK4b^), which are key regulators of cell proliferation. Upregulation of these cell cycle inhibitors is associated with GF senescence and impairment of ECM production (53, 54). Pathway analysis of DAC-induced genes also identified several processes related to interferon signaling and antiviral responses. While the functional consequences of this effect require further research, it should be noted that GF stimulation with bacterial cyclic dinucleotides results in the induction of a similar cluster of interferon signaling effectors, including *ISG15*, *MX1*, and *IFIT1* (55). Finally, several DAC-inducible genes in GFs overlap with the transcription profiles observed in gingival biopsies from patients with chronic periodontitis. Out of the 20 most upregulated genes in periodontitis tissues compared to controls (56), five were induced in DAC-treated GFs in our experiments (*CSF3*, *GLDC*, *SAA1*, *SAA2*, *GDF15*), highlighting the notion that DNMT inhibitors upregulate several genes that are associated with periodontitis pathology.

Although verification of the changes in DNA methylation profiles at individual gene promoters is beyond the scope of this study, many of the identified DAC-sensitive genes are reportedly regulated by changes in methylation of their promoter regions and sensitive to hypomethylating agents. Differential *CCL20* promoter methylation was identified in different T cell subsets (57) and in hepatocellular carcinoma (HCC) samples (58), correlating with mRNA expression levels. In line with our observations in GFs and PDLFs, *CCL20* expression was strongly induced by DAC treatment in HCC cells (58). Similarly, DAC-induced upregulation of *ICAM1* has been reported in several cancer cell lines, including cutaneous melanoma (59), pediatric sarcoma (60), and glioma cells (61). *MMP* genes are also regulated by promoter methylation (62–64). Interestingly, MMP1 is regulated by DNMT inhibitors in fibrosarcoma cells through a transcription-dependent mechanism that involves recruitment of the transcription factor Sp1 (65). This suggests that other mechanisms of action of hypomethylating agents may also be responsible for the observed induction of MMPs.

In our experiments, the chemokine CCL20 was highly susceptible to regulation by DNMT inhibitors. Because CCL20 shares common structural characteristics with β-defensins and its antimicrobial activity against many Gram-negative and Gram-positive bacteria has been confirmed *in vitro* (26), it was postulated that increased production of CCL20 may enhance innate immune responses by directly eliminating oral pathogens (66). We tested this possibility, and our analysis indicates that even at concentrations exceeding those released by DAC-treated GFs CCL20 is unable to affect *P. gingivalis* growth. It is also unlikely that local accumulation of CCL20 at high concentration will exert antimicrobial activity because CCL20 is rapidly degraded by *P. gingivalis* proteolytic enzymes. Consistently, *P. gingivalis* can potently degrade other chemokines with antimicrobial properties (28), such as CXCL10 (67). Instead, the amounts of CCL20 released by DAC-treated GFs are well within the range required for its chemotactic activity (26). Elevated production of CCL20 by cells treated with DNMT inhibitors may therefore contribute to the accumulation of leukocytes expressing CCR6, such as dendritic cells, B cells, and specific T cell subsets, including Th17 cells. It is likely that increased infiltration of CCR6-expressing leukocytes caused by augmented CCL20 release would contribute to exacerbation of chronic inflammation. However, formal verification of this possibility would require analysis of gingival tissue composition after DAC treatment in an animal model of periodontitis (68).

While global epigenomic studies are needed to characterize promoter-specific changes in DNA methylation profiles induced by DAC in stromal cells of the periodontium, these results show that hypomethylating agents are a useful tool to identify the genes and processes regulated by DNA methylation in cells involved in the pathogenesis of periodontitis. Conversely, the detrimental effects of DNMT inhibitors on GF and PDLF proliferation and viability, as well as the activation of multiple pro-inflammatory pathways associated with the pathobiology of periodontal disease, may greatly limit the therapeutic potential of these compounds.

## Data availability statement

The RNA-Seq dataset of DMSO- and DAC-treated GFs presented in this study has been deposited in the Gene Expression Omnibus (GEO) and is available under the accession number: GSE216757. 

## Ethics statement

The studies involving human participants were reviewed and approved by Bioethical Committee of the Jagiellonian University in Krakow, Poland (opinion number 1072.6120.104.2019) and the research ethics committee of the OLVG, Amsterdam, The Netherlands (protocol-ID: WO 17.194). The patients/participants provided their written informed consent to participate in this study.

## Author contributions

KBL-C and MM performed experiments and contributed to study design, data acquisition, analysis and interpretation. EN, AS, AB, and SD contributed to data acquisition. WL, MK, MT, DD, and MG contributed to recruitment of study participants, collection of samples and clinical data. TK, TJdV, and JP contributed to study conception and design. AMG conceived and designed the study, acquired funding, contributed to data analysis and interpretation, and drafted the manuscript. All authors contributed to manuscript revision, read and approved the submitted version.
