# Supplementary material for: Mapping of DNA methylation-sensitive cellular processes in gingival and periodontal ligament fibroblasts in the context of periodontal tissue homeostasis
Source: Front Immunol. 2023 Jan 26;14:1078031. doi: 10.3389/fimmu.2023.1078031 (PMC9909404; doi:10.3389/fimmu.2023.1078031)
Supplement: Supplementary file 1 [file DataSheet_1.pdf]

Supplementary Material

Supplementary Materials and Methods

Library preparation for mRNA sequencing

1 µg RNA per sample was used as input material for sample preparations. mRNA was enriched using oligo(dT) beads from NEBNext® Poly(A) mRNA Magnetic Isolation Module (cat# E7490L; NEB). Next, sequencing libraries were generated using a NEBNext Ultra II Directional RNA Library Prep Kit for Illumina® (cat# E7770L; NEB) according to the manufacturer's instructions. Briefly, fragmentation was performed using divalent cations under elevated temperature in NEBNext First Strand Synthesis Reaction Buffer (5X). First strand cDNA was synthesized using M-MuLV Reverse Transcriptase (RNaseH-) and random hexamer primer. Second strand cDNA was synthesized using DNA Polymerase I and RNase H and the reaction buffer containing dUTP instead of dTTP. The remaining overhangs were converted into blunt ends by exonuclease/polymerase activities. Following adenylation of 3' ends of DNA fragments, NEBNext Adaptor with hairpin loop structure were ligated to prepare for hybridization. The library fragments were purified with AMPure XP beads (cat# A63987; Beckman Coulter) to select cDNA fragments of 250-300 bp in length. Subsequently, 3 µl USER Enzyme (NEB) was incubated with size-selected, adaptor-ligated cDNA at 37° C for 15 min followed by 5 min incubation at 95°C. Next, PCR was performed using Phusion High-Fidelity DNA polymerase, Universal PCR primers and Index (X) Primer. Finally, PCR products were purified using AMPure XP beads and library quality was assessed using the Agilent High Sensitivity DNA Kit (cat# 5067-4626) on the Agilent Bioanalyzer 2100 system (Agilent Technologies).

Clustering and sequencing (Novogene Experimental Department)

The clustering of the index-coded samples was performed on a cBot Cluster Generation System (cat# SY401-2015, Illumina) using a TruSeq PE Cluster Kit v3-cBot-HS (cat# PE-401-3001, Illumia) as per the manufacturer's instructions. Following cluster generation, sequencing of the libraries was performed on an NovaSeq 6000 Illumina platform using a NovaSeq 6000 S2 Reagent Kit v1.5 cat. 20028314 -(300 cycles) and 150 bp paired-end reads were generated (minimum 6Gb and 20M).

Data processing and analysis

The quality of the raw reads was verified using the FastQC software (v. 0.11.9). Reads were aligned to the human reference genome GRCh38.p13 using the STAR software (v. 2.1.0). Gene expression levels were determined using the featureCounts tool (v. 2.0.3) and the GTF file from Ensembl database (v. 104) as a reference. All statistical analyses were performed using tools from the edgeR library (v. 3.34.0) with R software (v. 4.1.1). The raw counts were filtered using the filterByExpr function (with default parameters) and normalized using the TMM (Trimmed Mean of M values) method. Statistical analysis was then performed using the GLM method (according to the edgeR library documentation using the glmQLFit and glmQLFTest functions). The obtained p-values were corrected for multiple testing using the FDR method. Functional enrichment among genes with similar expression profiles was identified by using the Enrichr analysis tool (https://maayanlab.cloud/Enrichr/).

Supplementary Figures

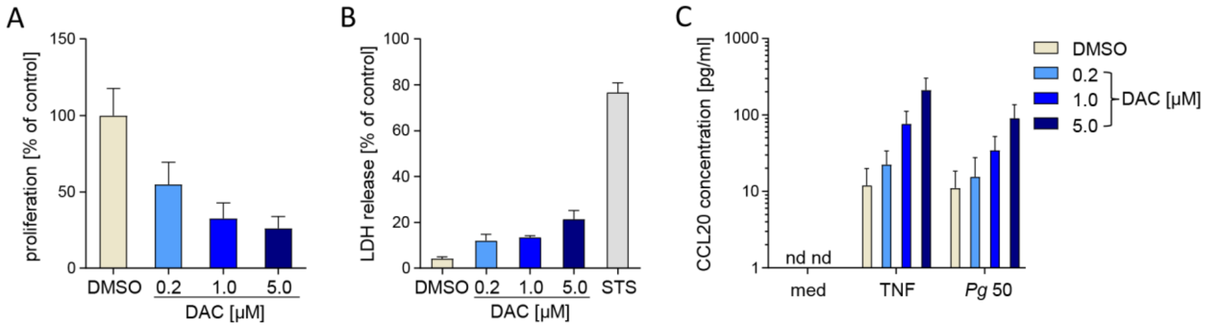

Figure S1. Dose-dependent effects of DAC on GF proliferation, viability, and CCL20 production. (A) Proliferation rate (n=4) and (B) LDH release (n=4) in GFs treated with DMSO or increasing concentrations of DAC (0.2, 1.0, 5.0 μM) for 12 days. (C) CCL20 production by DMSO- or DAC-treated GFs that were infected with *P. gingivalis* (MOI 50) for 1 h followed by 23 h of culture in fresh medium, or were stimulated with TNF (10 ng/ml) for 24 h (n=4-5).

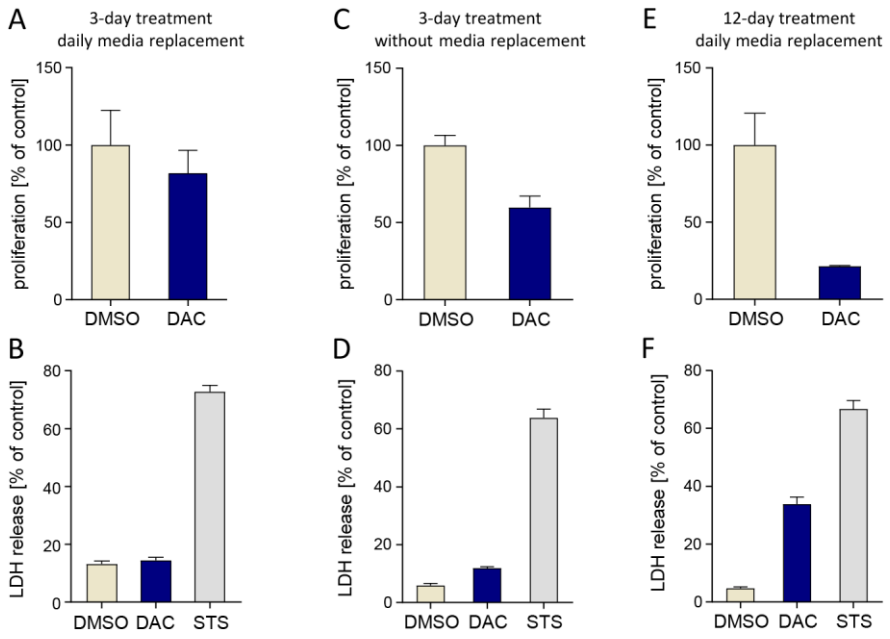

Figure S2. Comparison of DAC effects on GF proliferation and viability between different treatment protocols. Proliferation rate (A, C, E) and LDH release (B, D, F) by GFs cultured in the presence of DMSO or DAC: (A-B) for 3 days with replacement of medium and compounds every day, (C-D) for 3 days without replacement of medium and compounds, or (E-F) for 12 days with replacement of medium and compounds every day (n=2-4).

Supplementary Table

Table S1. Sequences of primers used for qPCR analyses.

Gene	Forward primer	Reverse primer
IL6	GACAGCCACTCACCTCTTCA	CCTCTTTGCTGCTTTCACAC
IL8	GCTCTGTGTGAAGGTGCAGT	CCAGACAGAGCTCTCTTCCA
CCL2	TCTGTGCCTGCTGCTCATAG	GGGCATTGATTGCATCTGGC
CCL20	AAGAGTTTGCTCCTGGCTGCTT	GCAGTCAAAGTTGCTTGCTGCT
COX2	AGCCCTTCCTCCTGTGCCT	AATCAGGAAGCTGCTTTTTACCT
MMP1	GGGAGATCATCGGGACAACTC	GGGCCTGGTTGAAAAGCAT
MMP9	CCTGGAGACCTGAGAACCAAT	GCCACCCGAGTGTAACCATAG
MMP13	AAGGAGCATGGCGACTTCT	TGGCCCAGGAGGAAAAGC
ICAM1	CCTTCCTCACCGTGTACTGG	AGCGTAGGGTAAGGTTCTTGC
TG2	CTGAGCACCAAGTACGATGC	GGACCCCTCTGGGTATTTGT
ITGB1	TGCGAGTGTGGTGTCTGTAA	AGGCTCTGCACTGAACACAT
RPLP0	GCGTCCTCGTGGAAGTGACATCG	TCAGGGATTGCCACGCAGGG
